# Supplementary material for: Autonomous nanorobots with powerful thrust under dry solid-contact conditions by photothermal shock
Source: Nat Commun. 2023 Nov 24;14:7663. doi: 10.1038/s41467-023-43433-6 (PMC10674020; doi:10.1038/s41467-023-43433-6)
Supplement: Supplementary file 1 — Supplementary Information [file 41467_2023_43433_MOESM1_ESM.pdf]

# Supplementary Information for

## Autonomous nanorobots with powerful thrust under dry solid-contact conditions by photothermal shock

**Zhaoqi Gu[1,4], Runlin Zhu[1,4], Tianci Shen[1,4], Lin Dou[1], Hongjiang Liu[1], Yifei Liu[1], Xu Liu[2], Jia Liu[3], Songlin Zhuang[1] & Fuxing Gu[1]***

*[1]Laboratory of Integrated Opto-Mechanics and Electronics, Shanghai Key Laboratory of Modern Optical System, School of Optical-Electrical and Computer Engineering, University of Shanghai for Science and Technology, Shanghai 200093, China.*

*[2]State Key Laboratory of Reliability and Intelligence of Electrical Equipment, Hebei University of Technology, Tianjin 300130, China.*

*[3]Department of Industrial and Systems Engineering, Auburn University, Auburn, AL 36849, USA.*

[4]These authors contributed equally: Zhaoqi Gu, Runlin Zhu, Tianci Shen.

*e-mail: gufuxing@usst.edu.cn

**The PDF file includes:**

Supplementary Note 1
Supplementary Figs. 1 to 10
Supplementary Table 1

## Supplementary Note 1. Why not photon momentum

We also notice the potential effects of photon momentum in physics, but it is not the driving mechanism in this wok, for which we give four reasons as following:

(i) We will first give numerical simulation evidences. With a typical beam waist radius of 4.3 μm, an average power of 1 μW, and repetition rate of 100 Hz, (instantaneous peak power 1 W, instantaneous light intensity $I$ = 1.7×10$^{10}$ W m$^{-2}$), we use the Lorentz formula to calculate the instantaneous optical force of a 10-μm-length gold nanowire and the net force is 1.7×10$^{-10}$ N, that is, 170 pN. This value is obviously much smaller than the μN force of friction that the nanowire is subjected to.

(ii) From the instantaneous power conversion of light momentum action, we can also consider the light pressure $P$, estimated with $P = 2rI/c$, where $I$ is the light intensity, $r$ is the reflectivity, and $c$ is the speed of light. Let $r = 1$ (full reflection) to get the maximum light pressure. According to the simulated data $I$ = 1.7×10$^{10}$ W m$^{-2}$, the optical pressure is calculated to be 56 Pa, while the Young's modulus of Au is 75 Gpa, so the strain is lower than 10$^{-11}$, which is equivalent to no deformation.

(iii) We can estimate the light gradient force by the following formula,

$$F_{ogrd} = \frac{2\pi n_0 a^3}{c}\left(\frac{m^2-1}{m^2+2}\right)\nabla I\ ,$$

where $n_0$ is the index of the target object, $a$ is the particle radius (characteristic size) and $m$ is the relative index between the object and background. We set $n_0$ = 0.54 (real part of index for Au in 532 nm), $m$ = 0.54, $a$ = 1 μm, and $I$ = 1.7×10$^{10}$ W m$^{-2}$. In Gaussian spot, the maximum light gradient occurs at half the radius of the beam waist. The maximum light gradient force is 10$^{-11}$ N, (if the characteristic size increases correspondingly, it may be increased to the magnitude order of pN). Many previous experiments have proven that for micro/nano-objects, the optical force provides forces of pN, while the adhesion force and friction force are in the magnitude order of μN. The pN force is sufficient to drive particles in fluid environments, but obviously not suitable in a solid interface. Strong adhesion and friction are the difficulties of solid interface driving.

In the previous three reasons, numerical simulation results were used to confirm that the light force is in the pN order. The thermal gradient force confirmed to be on the order of μN in the manuscript. We also provide experimental evidences for the next reason.

(iv) Silica is a commonly used material, without the photothermal effect in the wave band we used. We then used silica balls (4 μm and 8 μm in diameters), silica nanowires and silica nanoplates for comparative experiments, and we neither observed them being driven or damaged (melted). In this case, the light force, or the force generated by the photon momentum transfer, still exists, but does not work in driving. This excludes the possibility of pure optical driving.

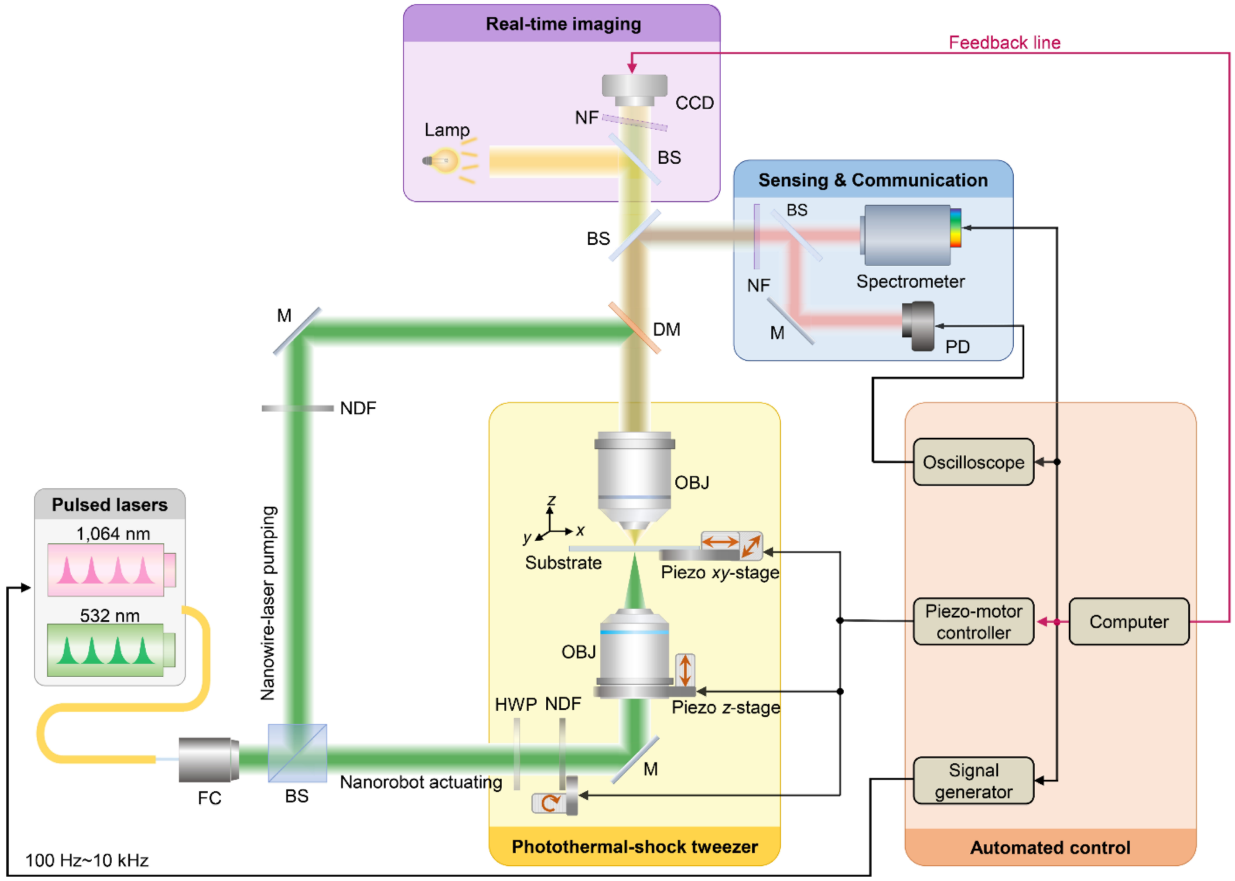


**Supplementary Figure 1**

**Experimental setup of actuation and motion control system.** The tweezer, imaging and sensing modules are all digitally controlled via homemade programs. The pulsed laser beam is divided into two beams by the BS: the lower beam is used to actuate nanorobots, and the upper beam is used to pump CdSe nanowires for sensing. FC: fibre coupler. M: mirror; NDF: neutral density filter; BS: beam splitter; HWP: half-wave plate; OBJ: object; DM: dichroic mirror; NF: notch filter; PD: photodetector.

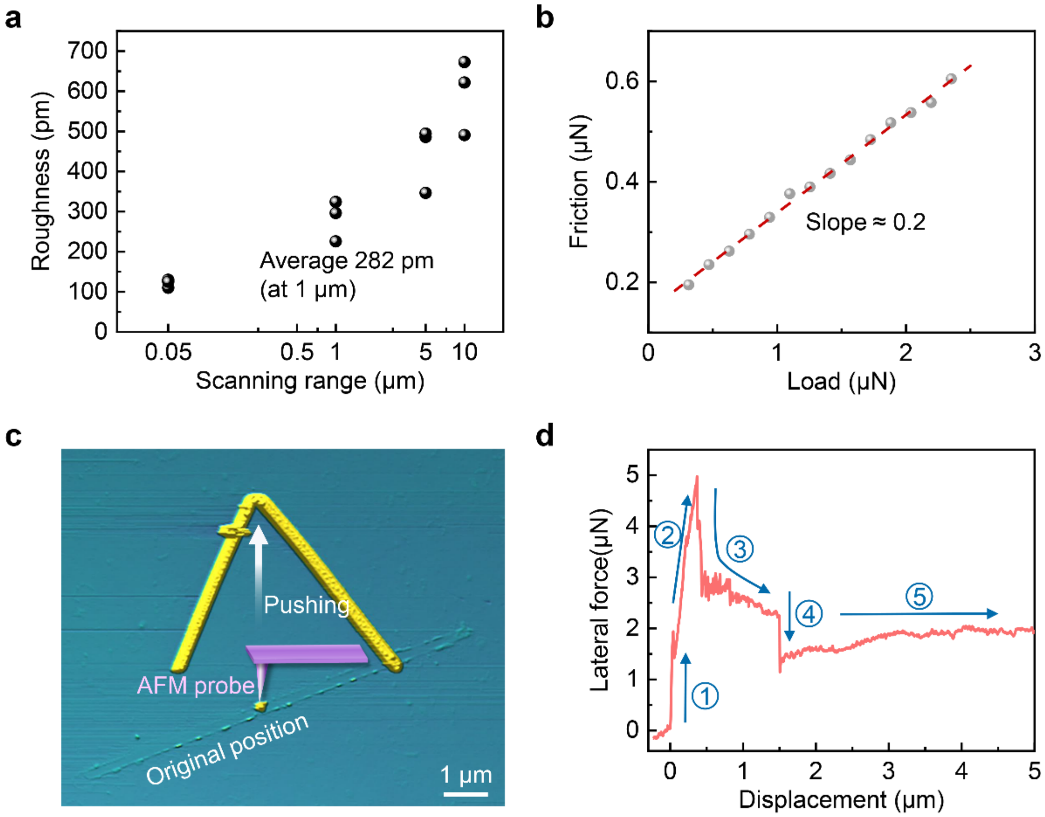


**Supplementary Figure 2**

**Surface characterization and friction measurement.** (**a**) Roughness scanning statistical graph of a typical silica substrate, according to the contact range between a gold nanowire and a silica substrate. We then chose a roughness value of 0.27 nm, close to the roughness average value (0.285 nm) at the scanning range of 1 μm. (**b**) Friction forces under different loads with a linear fitted slope of ~0.2. (**c**) AFM image of a bending nanowire, with a clear trace of its original position. The nanowire bent and locomoted over a distance of ~5 μm under an AFM probe pushing. (**d**) Displacement-dependent experimental results of probe-sensing lateral forces as pushing the nanowire in (c), which is divided into five processes: (1) The lateral force increased rapidly upon the AFM probe contact, while the nanowire remained stationary but it deformed; (2) The nanowire was pushed to overcome the maximum stiction force; (3) Lateral force suddenly decreased, as the entire nanowire was pushed and its stiction was completely transformed into dynamic friction; (4) The nanowire bent (or broken); (5) The entire nanowire locomoted over a distance of ~3 μm. Source data are provided as a Source Data file.

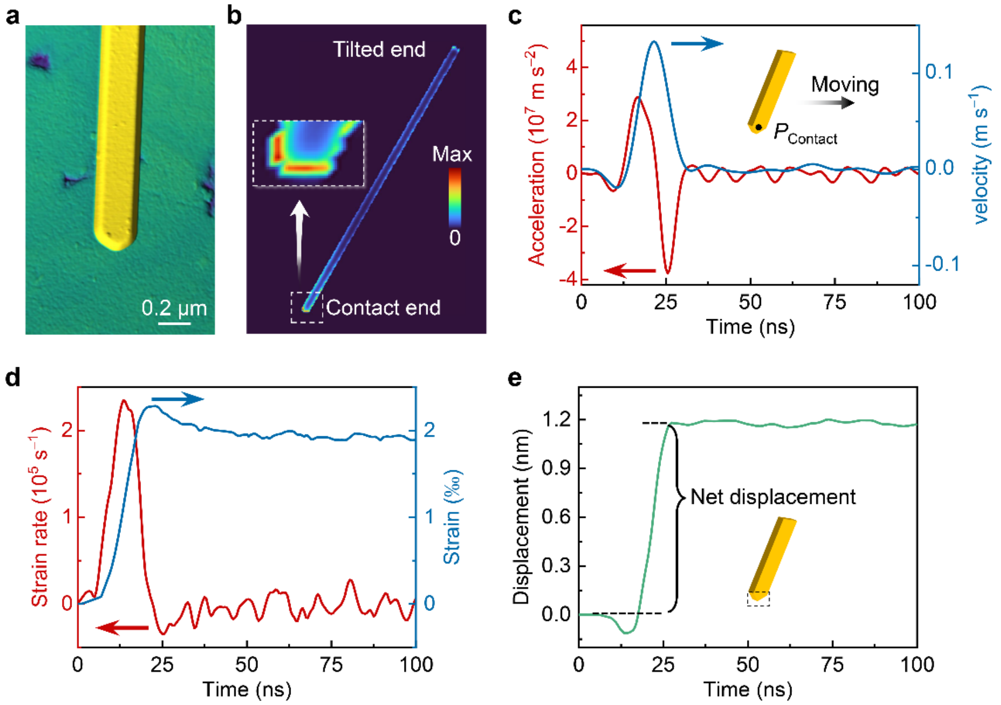


**Supplementary Figure 3**

**AFM characterization and simulation results for the tilted gold nanowire.** (**a**) AFM characterization of a typical nanowire end. (**b**) Normalized heat absorption factor distribution on the nanowire. Inset: Enlarged illustration near the contact end. (**c** to **e**) Time-dependent results of acceleration and velocity (c), strain rate and strain (d), and net displacement (e) (centroid displacement of the dashed region in inset) in the tilted nanowire. Such a high acceleration corresponds to an extremely large thrust-to-weight ratio of $2 \times 10^6$. The dimensional parameters in (b to e**)** are consistent with those in Fig. 1c (main text). The values in (c and d) are recorded from $P_{\text{Contact}}$ (7 nm away from the contact end) of the nanowire (c, inset). Source data are provided as a Source Data file.

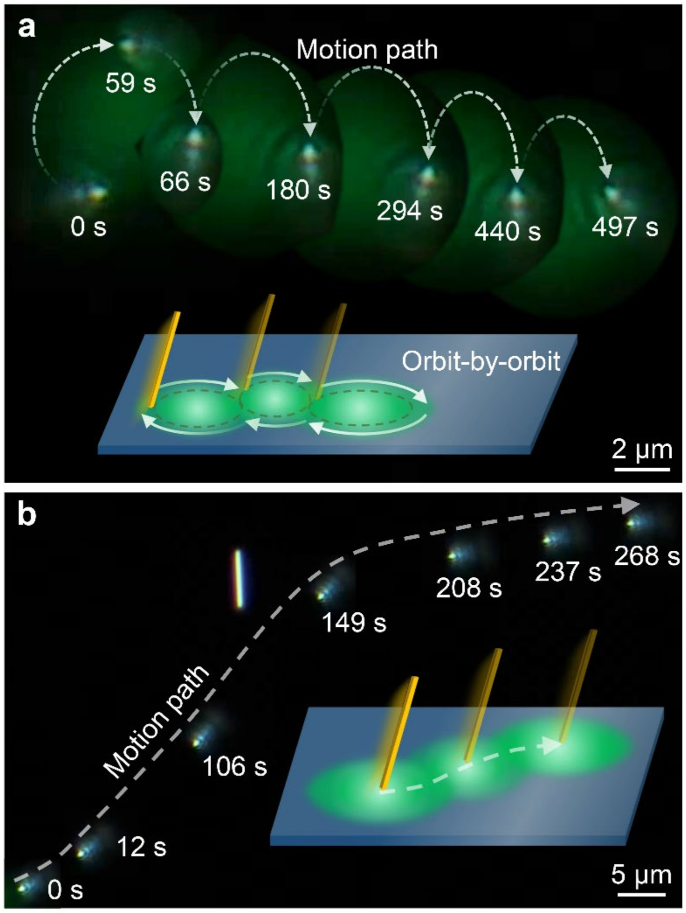


**Supplementary Figure 4**

**Motion control of tilted gold nanowires.** Sequential superimposed motion paths of transporting tilted nanowires. (**a**) A nanowire was transported to rotate along the iso-intensity lines (black dashed circles) by using an orbit-by-orbit method. Part of the spot edge is clipped so as not to obscure the nanowire. (**b**) A nanowire was repeatedly guided from the light spot centre to the edge (i.e., in the region with the highest gradient of light intensity) as the spot moving.

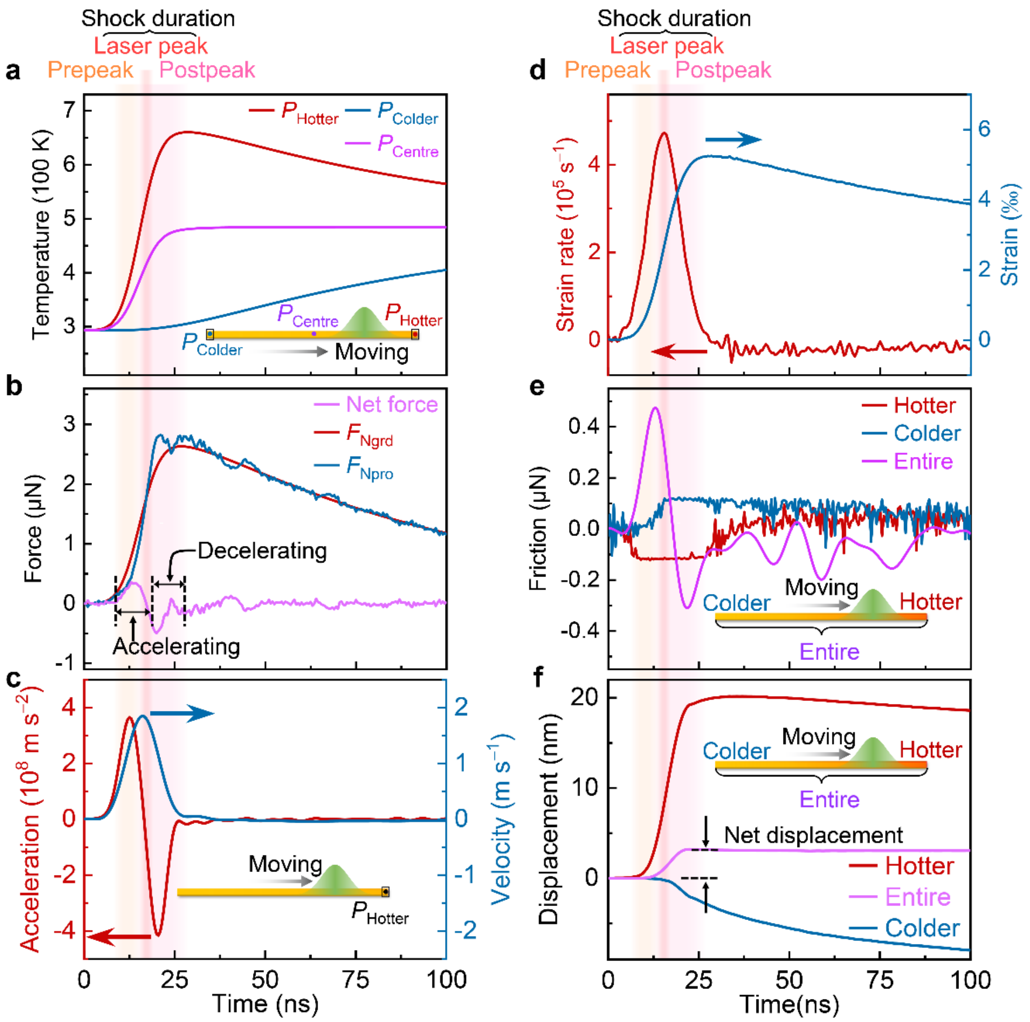


**Supplementary Figure 5**

**Time-dependent simulation results for the lying gold nanowire.** The dimensional parameters are consistent with those in Fig. 2b (main text). Thermal shock duration is divided into two stages of prepeak and postpeak, bounded by the laser peak (red line at 15th ns). Three typical points are selected for investigation: $P_{\mathrm{Hotter}}$ and $P_{\mathrm{Colder}}$ (distributed on the centres of the hotter and colder 1/32-length regions), and $P_{\mathrm{Centre}}$ (the entire nanowire centre). (**a**) Inconsistent temperature distribution, which lasts hundreds of nanoseconds. (**b**) Net force (integrated from the entire nanowire) is the difference between $F_{\mathrm{Ngrd}}$ and $F_{\mathrm{Npro}}$, and causes accelerations (prepeak) and decelerations (postpeak). Thermal gradients (a) and thermal gradient forces (b) are always there, but the net forces exist only during thermal shock. (**c** and **d**) Acceleration and strain rate recorded from $P_{\mathrm{Hotter}}$, corresponding to a huge thrust-to-weight ratio of $4 \times 10^7$. (**e** and **f**) Friction force and centroid displacement, which are integrated from the hotter and colder 1/32-length regions, respectively. The net force in (b) is numerically equal to the entire friction force in (e). Source data are provided as a Source Data file.

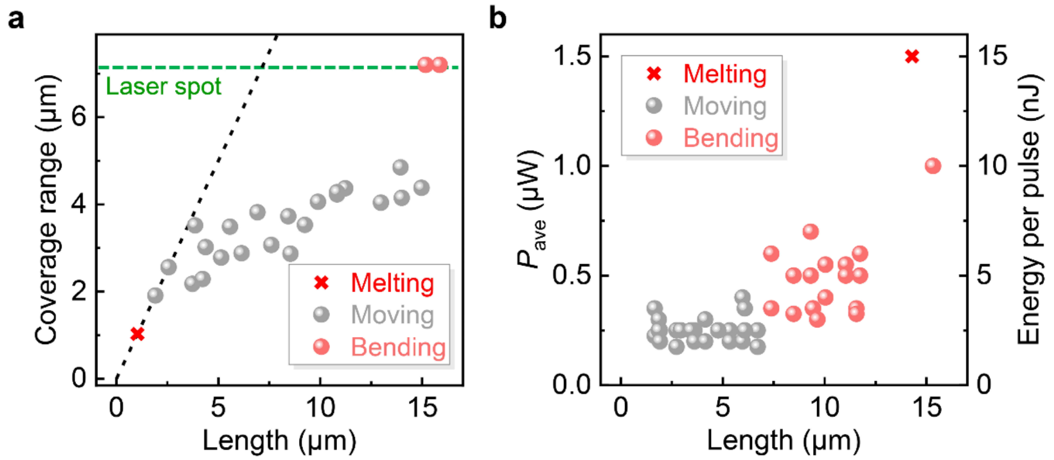


**Supplementary Figure 6**

**Statistical results of minimum light coverage and power to actuate different gold nanowires.** For a constant light spot with $D_{spot}$ = 7.2 μm, the required minimum light coverage (**a**) and minimum $P_{ave}$ (**b**) are provided. The green and black dashed lines in (a) denote the coverage ranges equal to $D_{spot}$ and the nanowire length. Short nanowires with lengths less than ~1 μm were difficult to move, even $P_{ave}$ was too high to make them melted. The axial motion control requires only partial light coverage of the nanowires, while the lateral motion control requires a higher proportion, even a complete coverage. Source data are provided as a Source Data file.

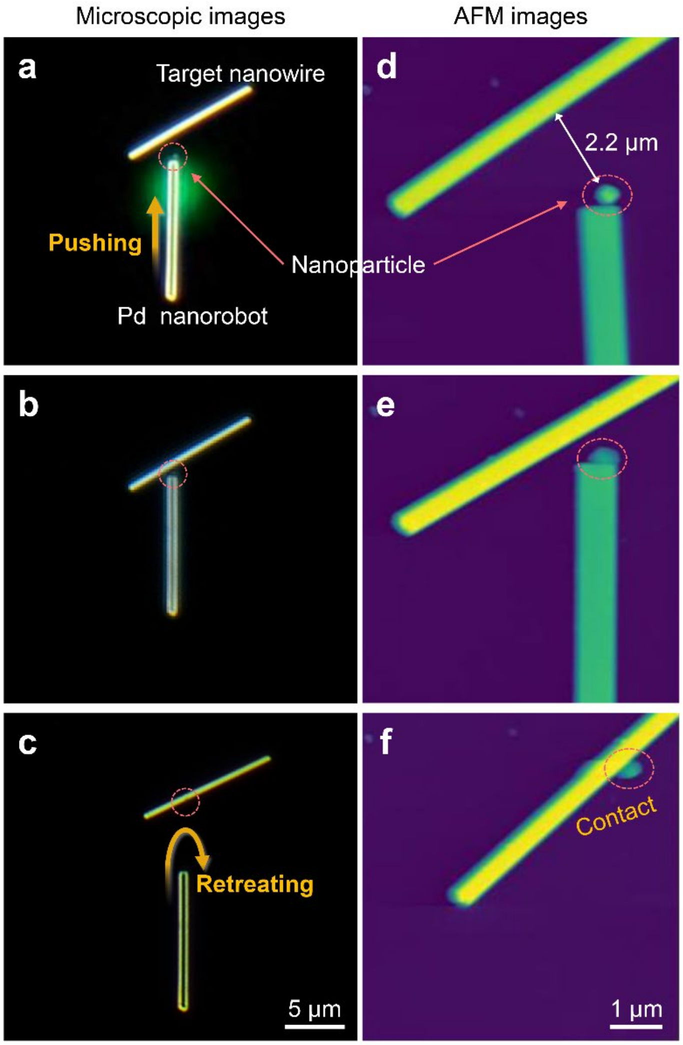


**Supplementary Figure 7**

**Assembly of an optical near-filed coupling structure using a Pd nanowire nanorobot.** Initially, (**a** and **d**) a Pd nanowire nanorobot was driven by the light spot, then (**b** and **e**) pushed a Pd nanoparticle (~100 nm in diameter) until (**c** and **f**) the nanoparticle contacted the target nanowire, and finally retreated for clearly AFM scanning, forming a nanoparticle-nanowire optical near-filed coupling structure. Red dashed circles indicate the locations of the nanoparticles.

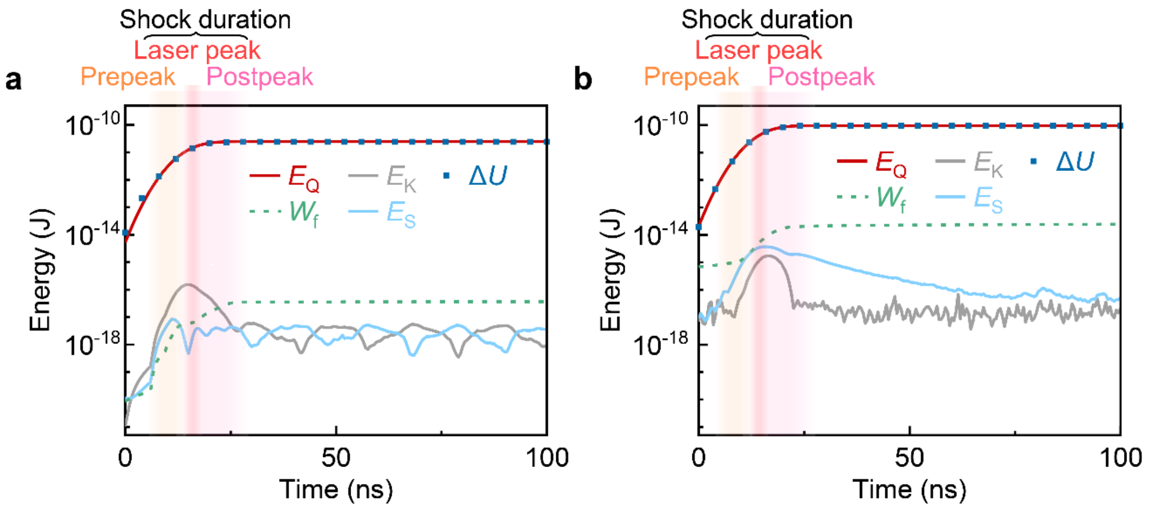


**Supplementary Figure 8**

**Time-dependent energy during thermal shock for tilted and lying gold nanowires.** Time variations of energy for the tilted nanowire (**a**, corresponding to Fig. 1c in the main text) and the lying nanowire (**b**, corresponding to Fig. 2b in the main text). The values of $E_k$ in both nanowires increase rapidly during thermal shock, and both account for about $10^{-4}$ of $E_Q$. $E_Q$: total absorbed thermal energy; $E_K$: total kinetic energy; $E_S$: total strain energy (elastic potential energy); $\Delta U$: total increased internal energy; $W_f$: total friction-loss energy. Source data are provided as a Source Data file.

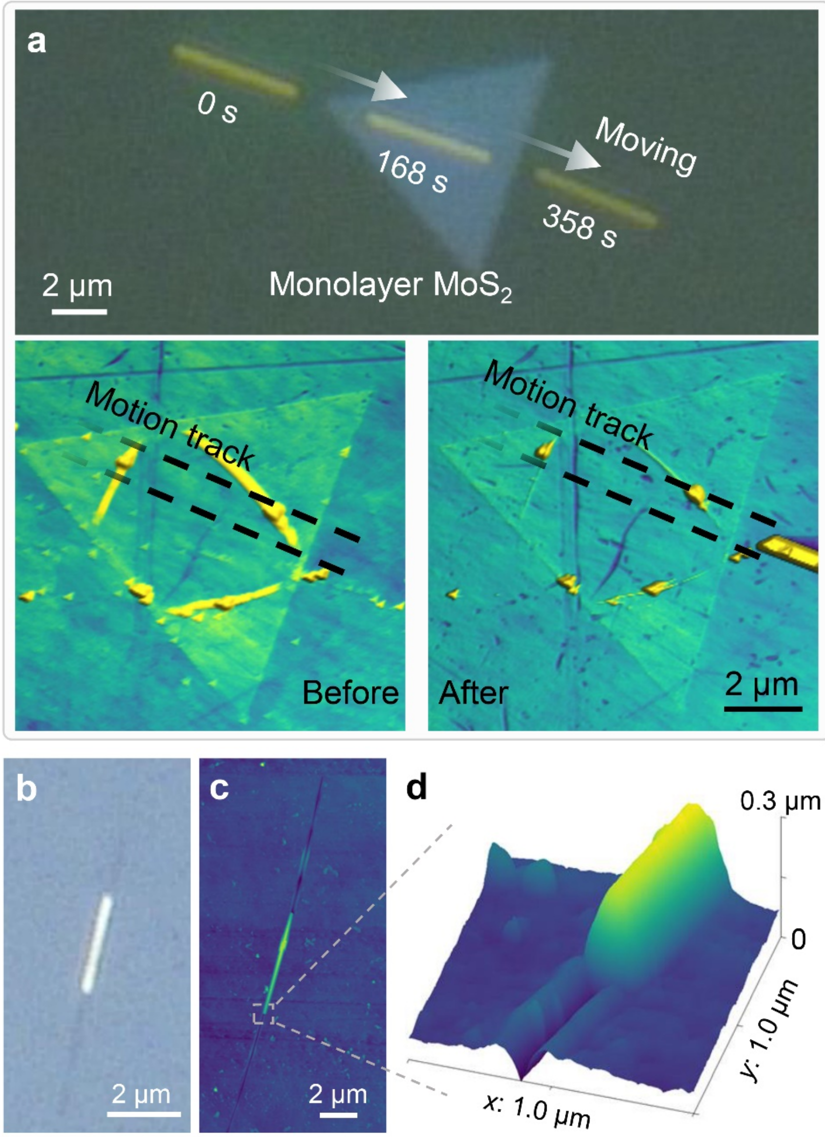


**Supplementary Figure 9**

**Different substrates for the photothermal shock driving.** (**a**) Superimposed photographs of a gold nanowire sliding across a monolayer MoS2 on a silica substrate at three sequential moments. (Bottom) Comparison of high-resolution AFM images of surface changes on the monolayer before and after nanowire sliding. Optical (**b**) and AFM (**c**) images for a gold nanowire on a polystyrene substrate, and the enlarged AFM 3D image (**d**). Visible scratches are observed under the optical microscope on the polystyrene substrate (b), and the AFM surface characterization reveals that the axis of the nanowire delineated a channel with a width of ~80 nm and a depth of ~30 nm (c and d). The channel on the polystyrene substrate comes from thermal damage.

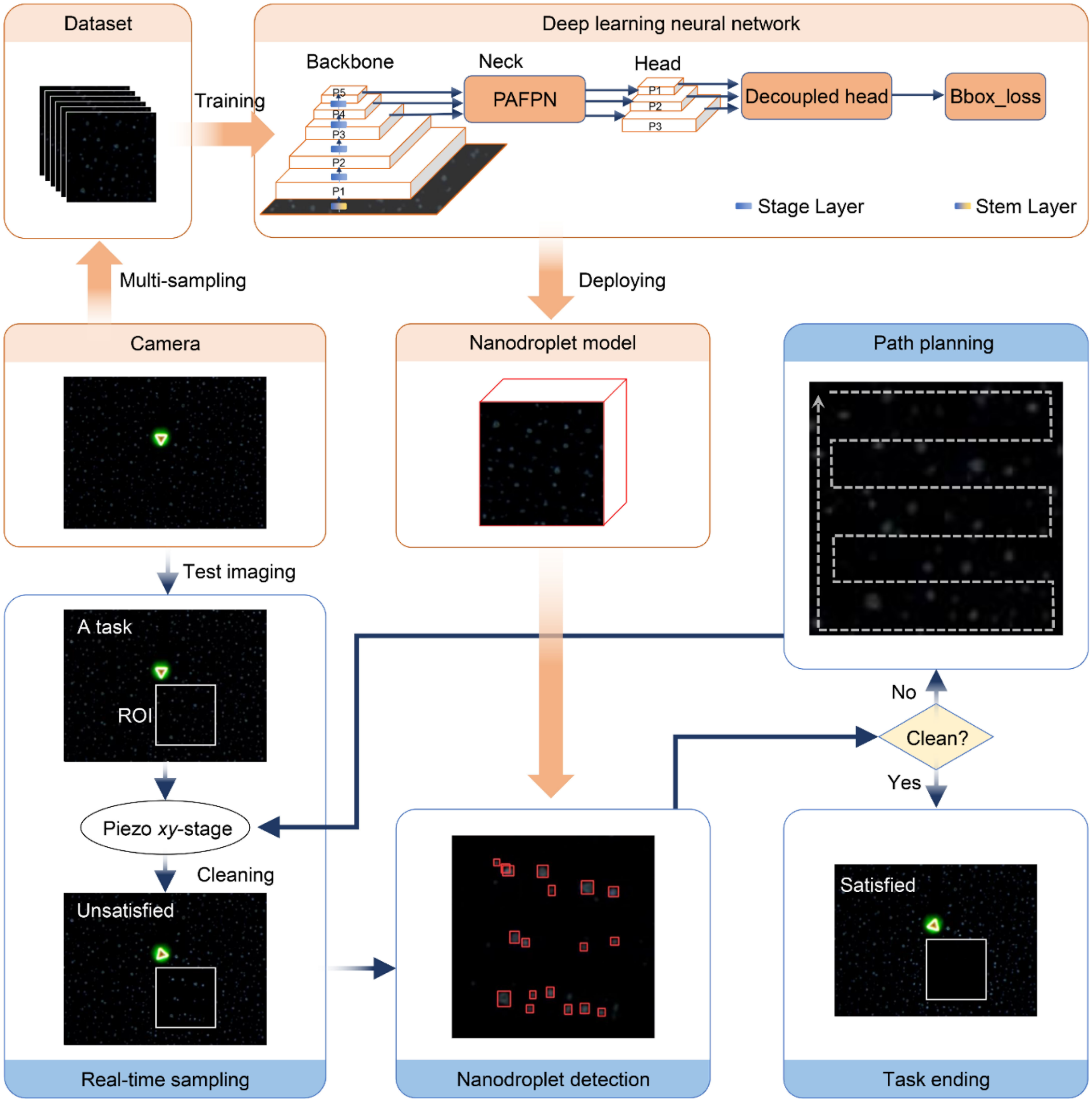


**Supplementary Figure 10**

**Autonomous control system of the cleaning nanorobot.** The camera sampled enough images as deep learning training samples to generate a nanodroplet model for image recognition, and also performed real-time sampling during a cleaning task of the nanorobot. Once the program started, the nanorobot automatically performed the cleaning cycle, including path planning, cleaning and image recognition, until satisfactory cleanliness was achieved (no nanodroplets detected). The class scan path interval for each new cycle was halved from the previous cycle. PAFPN: Path aggregation feature pyramid network.

**Supplementary Table 1. Values of parameters used in simulations.**

| Parameter | Definition | Value/unit |
|---|---|---|
| $\rho$ | Density of gold | 19.3 g·cm$^{-3}$ |
| $t_0$ | Time delay of the pulse peak | 60 ns |
| $t_p$ | Duration of the pulsed laser | 10 ns |
| $\tau_0$ | Phonon relaxation time of gold | 35 ps |
| $c_p$ | Specific heat capacity of gold | 129 J·kg$^{-1}$·K$^{-1}$ |
| $k$ | Thermal conductivity of gold | 318 W·m$^{-1}$·K$^{-1}$ |
| $E$ | Young's modulus of gold | 75 GPa |
| $v$ | Poisson's ratio of gold | 0.44 |
| $\alpha$ | Thermal expansion coefficient of gold | 14.2 × 10$^{-6}$ K$^{-1}$ |
| $n_m + in_k$ | Index of gold at 532-nm wavelength | 0.54 + 2.23i |
| $n_d$ | Index of silica | 1.45 |
