# Supplementary material for: Autonomous nanorobots with powerful thrust under dry solid-contact conditions by photothermal shock
Source: Nat Commun. 2023 Nov 24;14:7663. doi: 10.1038/s41467-023-43433-6 (PMC10674020; doi:10.1038/s41467-023-43433-6)
Supplement: Supplementary file 3 — Description of Additional Supplementary Files [file 41467_2023_43433_MOESM3_ESM.pdf]

Supplementary Movie 1

Description: Motion control of tilted nanowires. (1) A tilted gold nanowire quickly left the light spot centre and then moved clockwise in a quasi-circular motion. (2) The nanowire was transported by using an orbit-by-orbit method, rotating along the iso-intensity lines (black dashed circles). (3) Another tilted nanowire was directly pushed forward by the spot, leaving the spot repeatedly. P_{ave} was gradually increased from 0.5 μW to 1.0 μW in this section.

Supplementary Movie 2

Description: Dynamic simulation of a tilted nanowire. The nanowire size is consistent with that in Fig. 1c (main text). The nanowire only slides within the thermal shock duration. The tilted end remains vibrating after the thermal shock, while the contact end remains stationary with the substrate.

Supplementary Movie 3

Description: Axial motion control. (1) A gold nanowire moved into the light spot until their centres roughly coincided (i.e., the nanowire was trapped by the spot). (2) Another gold nanowire was manipulated axially by a light spot over a distance of ~30 μm.

Supplementary Movie 4

Description: Dynamic simulation of a lying nanowire. The nanowire size is consistent with that in Fig. 2b (main text). The centroid of the entire nanowire moves towards the hotter end due to the extreme expansion in the hotter end during thermal shock, but does not change after the thermal shock.

Supplementary Movie 5

Description: Separation of a seven-nanowire cluster. A seven-gold-nanowire cluster was separated into individual nanowires by using a pulsed light excitation. Therefore, thermal shock can also weaken the adhesion force between nanomaterials.

Supplementary Movie 6

Description: Nanowires bouncing off the substrates. The tilted nanowire in (1) and the lying nanowire bounced off the silica substrate, and disappeared, while the tilted nanowire in (3) bounced off the silica substrate, and returned.

Supplementary Movie 7

Description: Lateral motion control. (1) A gold nanowire that was originally in a light spot centre bent into a bow which roughly coincided with an iso-intensity line near the spot edge. (2) Another gold nanowire was manipulated to bend and move laterally by a light spot over a distance of ~30 μm.

Supplementary Movie 8

Description: Driving a silver nanowire. A silver nanowire was driven by a 532-nm-wavelength pulsed light, which proves the generality of photothermal shock to target materials.

Supplementary Movie 9

Description: Annular-shape light spot. The gold nanowire and nanoplate was trapped in the annular sector, rather than the dark centre, because the maximum light intensity is in the centre of the annular sector. The annular-shaped light was generated by an optical vortex phase plate.

Supplementary Movie 10

Description: Thrust output. A gold nanowire was first driven by the light spot, and then the nanowire output an extra thrust to laterally push another nanowire bending into an acute angle. Finally, two nanowires formed an arrow shape.

Supplementary Movie 11

Description: Cleaning nanorobot. The cleaning nanorobot (nanoplate) performed three-cycle cleaning under image recognition and feedback control. After the third cleaning cycle, the nanorobot stopped working as the cleanliness was satisfied.

Supplementary Movie 12

Description: HOUbot motion control. (1) The HOUbot circled around a target object. (2) The HOUbot first moved straight forward, then reversed and returned to the initial position. The HOUbot performed head pushing (3), tail wagging (4), and tail stabbing (5).
